# Supplementary material for: Valency-Limited Molecular Dynamics Simulations of Stickers-and-Spacers Polymers Reveal a Tradeoff Between Condensation and Organization
Source: bioRxiv. 2026 Aug 6:2026.08.02.742325. Preprint. [Version 1] doi: 10.64898/2026.08.02.742325 (PMC13484616; doi:10.64898/2026.08.02.742325)
Supplement: Supplementary file 1 [file media-1.pdf]

Valency-Limited Molecular Dynamics Simulations of Stickers-and-Spacers Polymers Reveal a Tradeoff Between Condensation and Organization

Yumeng Zhang,[†,‡] Amogh Sood,[†,‡] Advait Athreya,[†] and Bin Zhang[*,†]

†*Department of Chemistry, Massachusetts Institute of Technology, Cambridge, MA, USA*

‡*Contributed equally to this work*

E-mail: binz@mit.edu

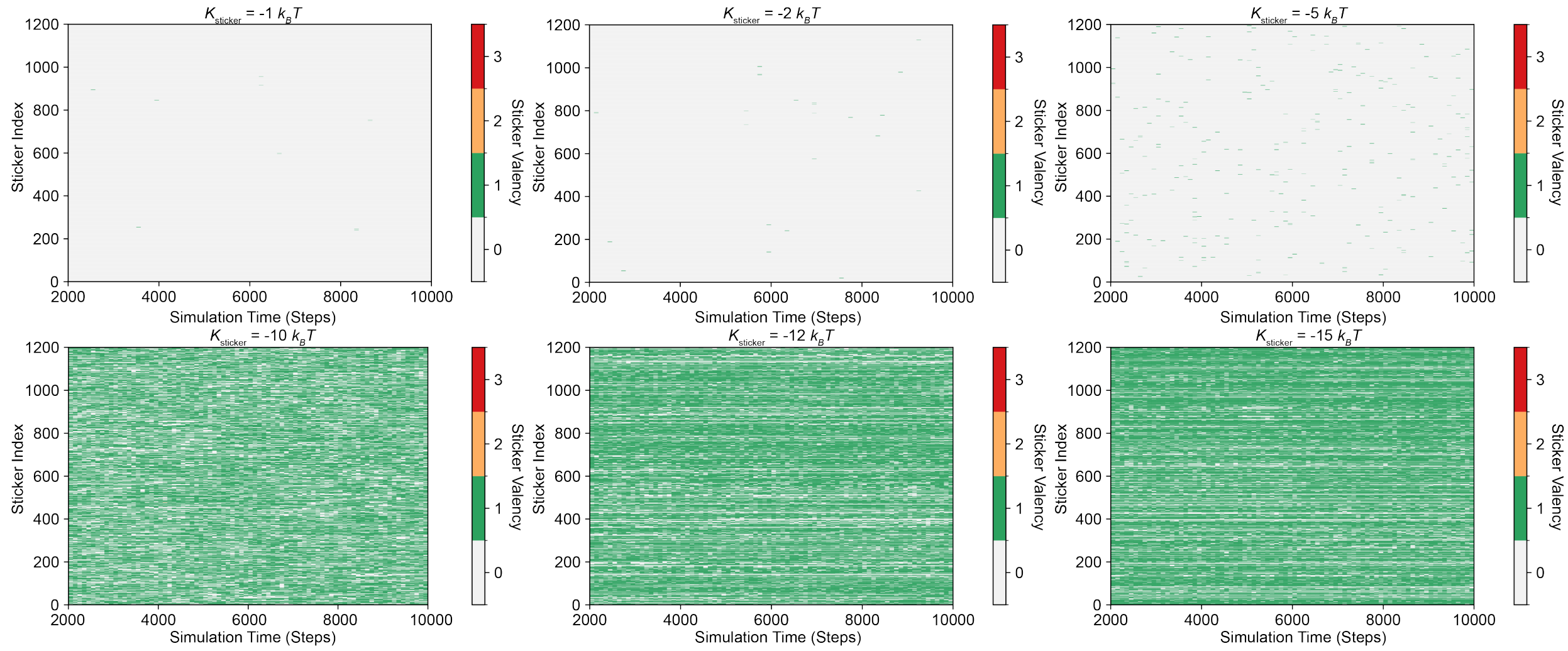

Figure S1: **Time-resolved sticker valency at different sticker interaction strengths.** Each row corresponds to an individual sticker, indexed on the y-axis, and the x-axis denotes simulation time. Color indicates the instantaneous sticker valency, $v_i(t)$, defined as the number of other stickers bound to sticker i at time t as in Eq. 10 of the main text. The trajectories are the same as those analyzed in Figure 3A.

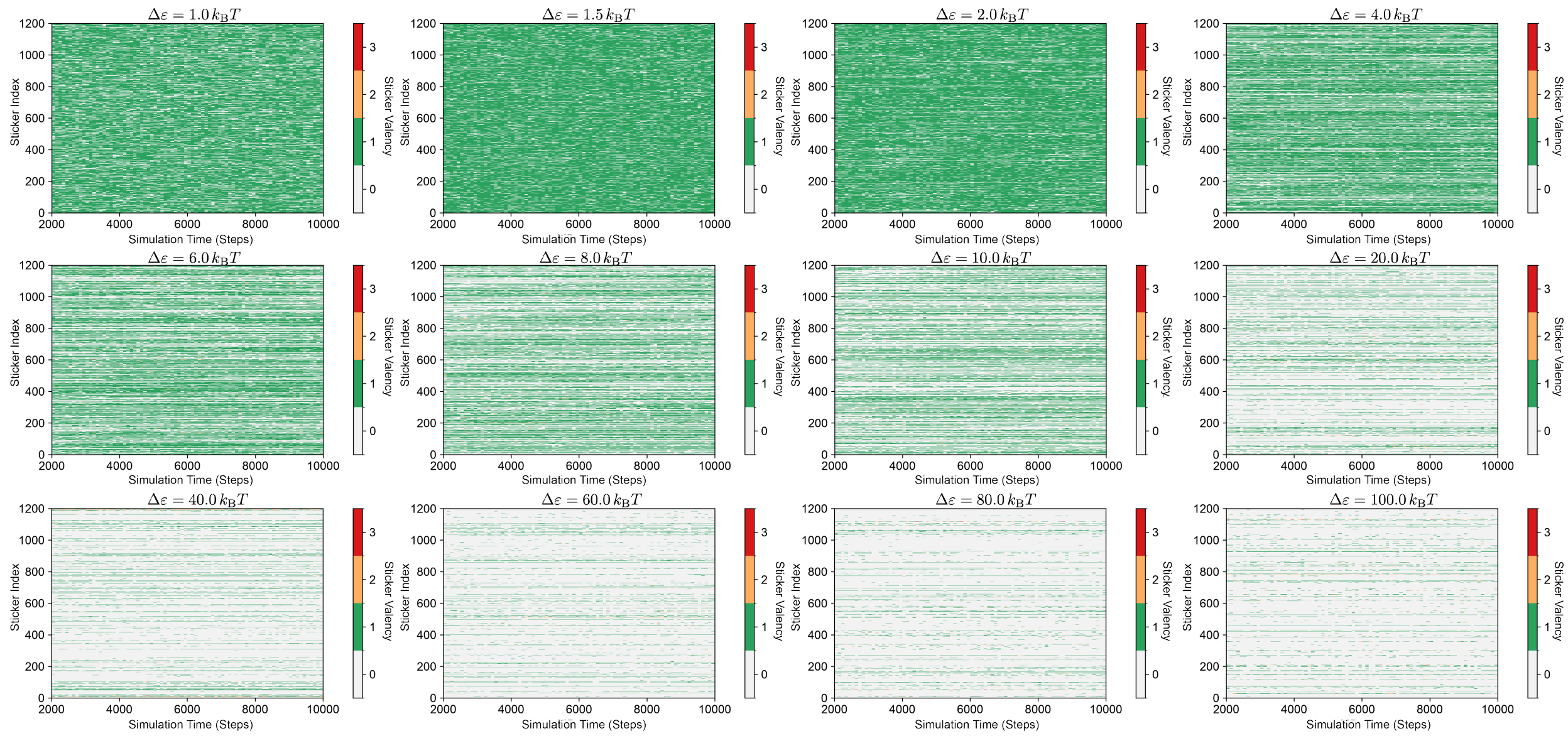

Figure S2: **Time-resolved sticker valency at different levels of spacer-interaction heterogeneity.** Each row corresponds to an individual sticker, indexed on the y-axis, and the x-axis denotes simulation time. Color indicates the instantaneous sticker valency, $v_i(t)$, defined as the number of other stickers bound to sticker i at time t as in Eq. 10 of the main text. The trajectories are the same as those analyzed in Figure 3B.

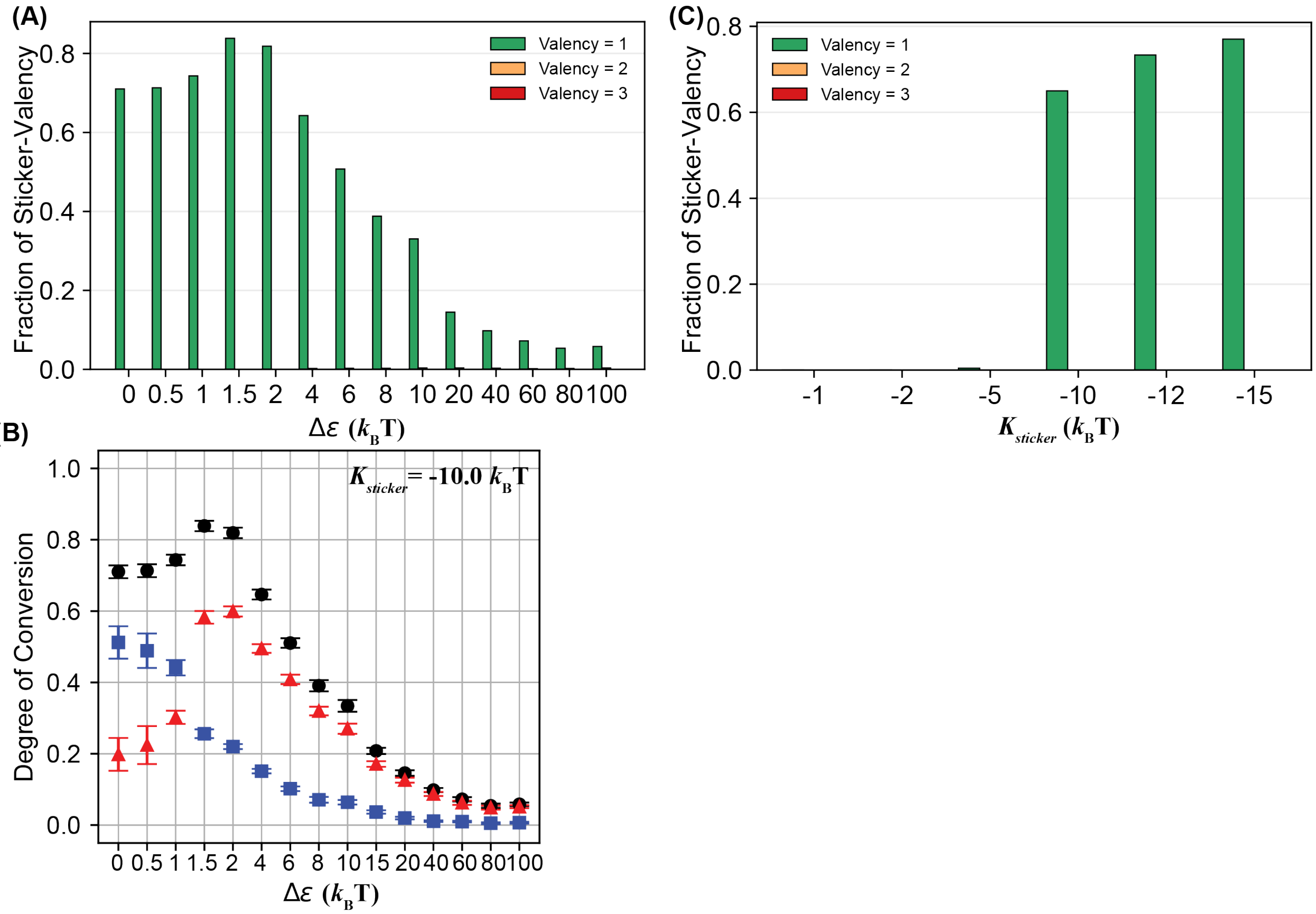

Figure S3: **Sticker valency statistics and degree of conversion for one-component systems.** (A) Fraction of bound sticker valency and (B) degree of conversion as a function of $\Delta\epsilon$, at fixed $K_{\text{sticker}} = -10\, k_{\text{B}}T$ and $\bar{\epsilon} = 0\, k_{\text{B}}T$. The fraction of unbound stickers (valency = 0) was not presented for clarity. (C) Fraction of sticker valency as a function of K_{sticker} at fixed $\bar{\epsilon} = 0\, k_{\text{B}}T$ and $\Delta\epsilon = 0\, k_{\text{B}}T$. The corresponding degree of conversion analysis is shown in Figure 3A.

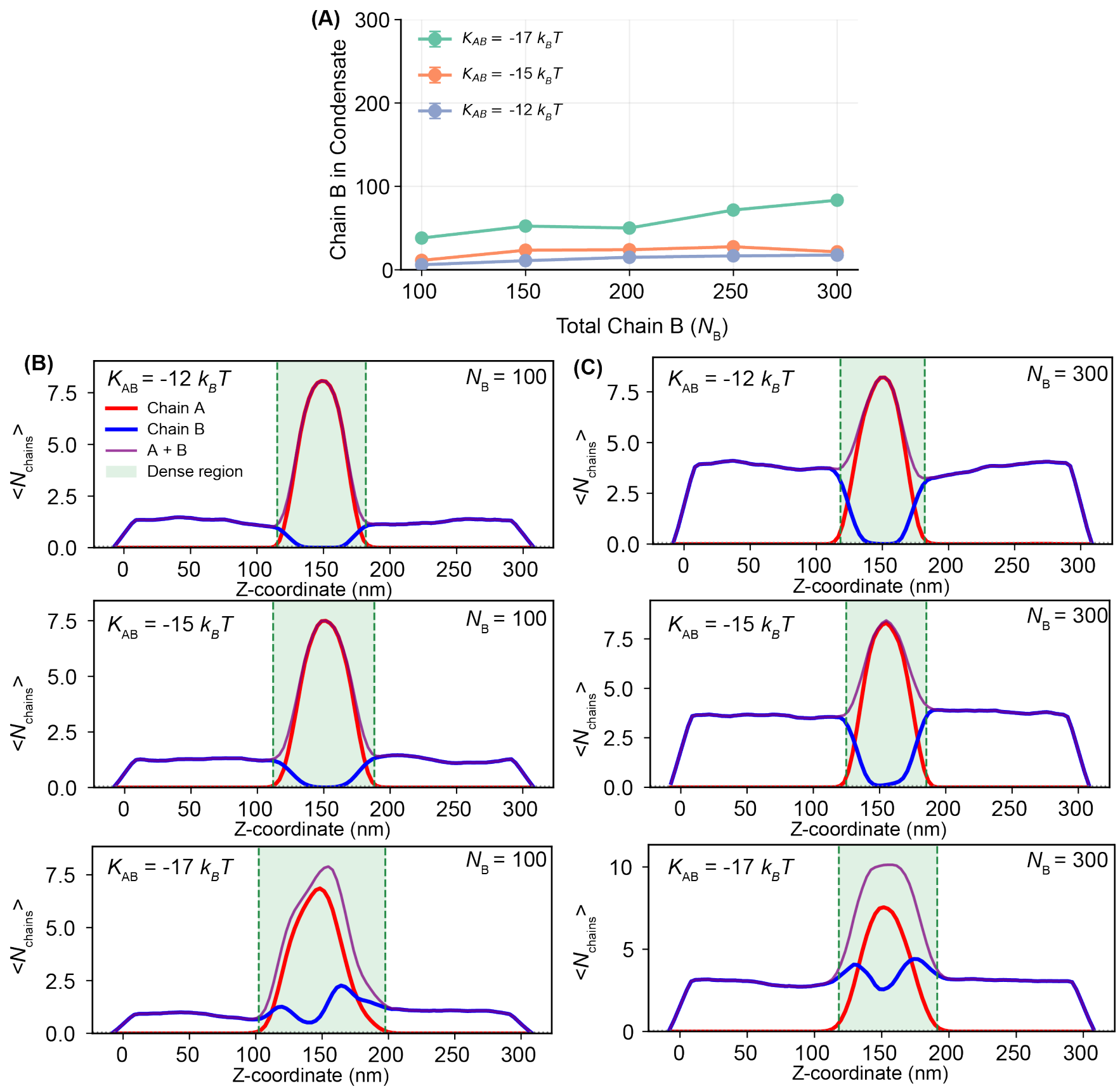

Figure S4: **The saturable recruitment observed in Figure 4A is robust to changes in sticker interaction strength.** (A) Number of B molecules recruited into the A condensate as a function of the total number of B molecules in the system for sticker-mediated A–B interactions. Simulations were performed using the same protocol as in Figure 4A but with different A–B sticker interaction strengths. The curve for $K_{\mathrm{AB}} = -17\,k_{\mathrm{B}}T$ is identical to that shown in Figure 4A. (B–C) Representative density profiles for systems with $N_{\mathrm{B}} = 100$ and $N_{\mathrm{B}} = 300$ at different A–B sticker interaction strengths.

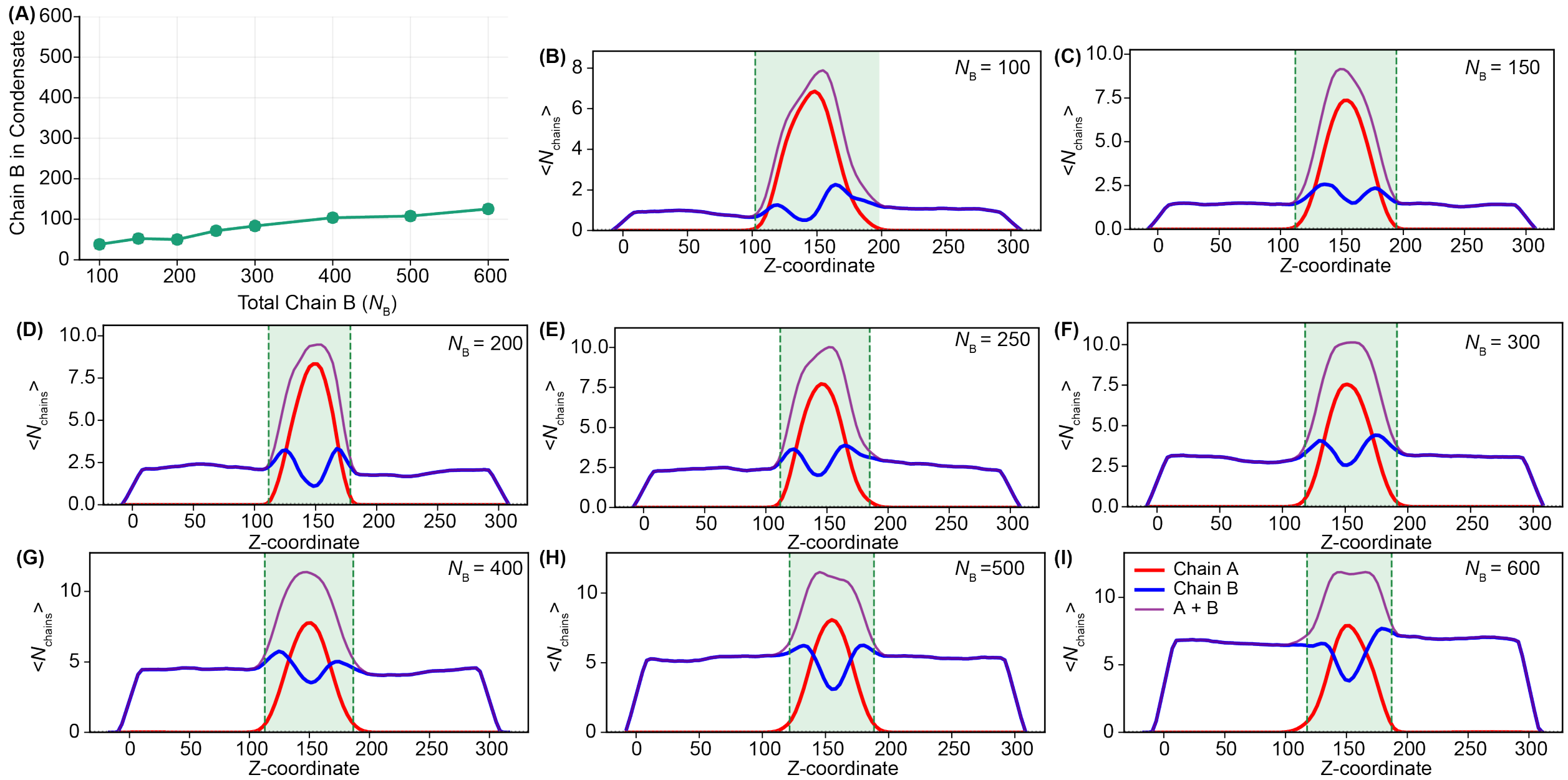

Figure S5: **Specific sticker interactions buffer client recruitment in two-component condensates.** (A) Number of B molecules recruited into the A condensate as a function of the total number of B molecules in the system for sticker-mediated A–B interactions. The data for $N_B \leq 300$ are presented in Figure 4A. (B-I) Density profiles for the simulations presented in panel A.

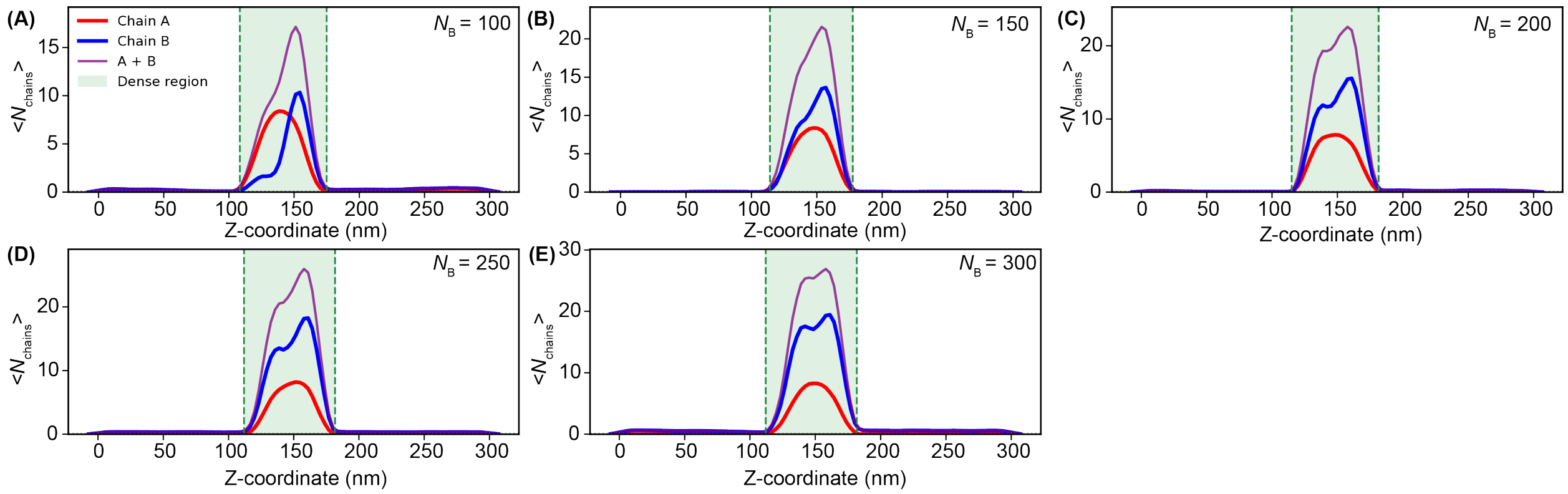

Figure S6: **(A-E) Density profiles for simulations presented in Figure 4C.**

Table S1: **Explicit architectures of chain A and chain B polymers.** All backbone beads are spacers. Auxiliary sticker beads are shown under their parent backbone bead. For A–A interactions and the one-component valency analysis, the default selector uses the even-numbered auxiliary stickers on Chain A, $S_2, S_4, \ldots, S_{24}$, corresponding to 12 active stickers per A chain. This default was used for the one- and two-component systems unless otherwise specified. For Figure 2B, the A–A selector was varied by `[::12]`,`[::6]`, `[::4]`, `[::3]`, `or [::2]` (every 12th, 6th, 4th, 3rd, or 2nd auxiliary sticker) to obtain $N_{\text{sticker}} = 2, 4, 6, 8$, or 12 active stickers per Chain A. For A–B interactions, the complementary odd-numbered stickers on Chain A, $S_1, S_3, \ldots, S_{23}$, interact with all four sticker beads on Chain B.

**Chain A: spacer positions 1–25**																									
Position	1	2	3	4	5	6	7	8	9	10	11	12	13	14	15	16	17	18	19	20	21	22	23	24	25
Spacer	A_1	A_2	A_3	A_4	A_5	A_6	A_7	A_8	A_9	A_{10}	A_{11}	A_{12}	A_{13}	A_{14}	A_{15}	A_{16}	A_{17}	A_{18}	A_{19}	A_{20}	A_{21}	A_{22}	A_{23}	A_{24}	A_{25}
Auxiliary sticker				S_1			S_2			S_3			S_4			S_5			S_6			S_7			S_8
Selected for A–A binding							S_2						S_4						S_6						S_8
Selected for A–B binding (A side)				S_1						S_3						S_5						S_7			

**Chain A: spacer positions 26–50**																									
Position	26	27	28	29	30	31	32	33	34	35	36	37	38	39	40	41	42	43	44	45	46	47	48	49	50
Spacer	A_{26}	A_{27}	A_{28}	A_{29}	A_{30}	A_{31}	A_{32}	A_{33}	A_{34}	A_{35}	A_{36}	A_{37}	A_{38}	A_{39}	A_{40}	A_{41}	A_{42}	A_{43}	A_{44}	A_{45}	A_{46}	A_{47}	A_{48}	A_{49}	A_{50}
Auxiliary sticker			S_9			S_{10}			S_{11}			S_{12}			S_{13}			S_{14}			S_{15}			S_{16}	
Selected for A–A binding						S_{10}						S_{12}						S_{14}						S_{16}	
Selected for A–B binding (A side)			S_9						S_{11}						S_{13}						S_{15}				

**Chain A: spacer positions 51–74**																								
Position	51	52	53	54	55	56	57	58	59	60	61	62	63	64	65	66	67	68	69	70	71	72	73	74
Spacer	A_{51}	A_{52}	A_{53}	A_{54}	A_{55}	A_{56}	A_{57}	A_{58}	A_{59}	A_{60}	A_{61}	A_{62}	A_{63}	A_{64}	A_{65}	A_{66}	A_{67}	A_{68}	A_{69}	A_{70}	A_{71}	A_{72}	A_{73}	A_{74}
Auxiliary sticker		S_{17}			S_{18}			S_{19}			S_{20}			S_{21}			S_{22}			S_{23}			S_{24}	
Selected for A–A binding					S_{18}						S_{20}						S_{22}						S_{24}	
Selected for A–B binding (A side)		S_{17}						S_{19}						S_{21}						S_{23}				

**Chain B: spacer positions 1–14**														
Position	1	2	3	4	5	6	7	8	9	10	11	12	13	14
Spacer	B_1	B_2	B_3	B_4	B_5	B_6	B_7	B_8	B_9	B_{10}	B_{11}	B_{12}	B_{13}	B_{14}
Auxiliary sticker				T_1			T_2			T_3			T_4	
Selected for A–B binding (B side)				T_1			T_2			T_3			T_4	
